# Birth preparedness, complication readiness and male partner involvement for obstetric emergencies in rural Rwanda

**DOI:** 10.11604/pamj.2016.25.91.9710

**Published:** 2016-10-17

**Authors:** Richard Kalisa, Oliver Ombeva Malande

**Affiliations:** 1 Department of Obstetrics and Gynecology, Ruhengeri Hospital, Musanze, Rwanda; 2 Athena Institute for Research on Innovation and Communication in Health and Life Sciences, VU University, Amsterdam, The Netherlands; 3 Department of Pediatrics, Faculty of Health Sciences, Egerton University, Nakuru, Kenya

**Keywords:** Antenatal care, barriers to access, male partner involvement, pregnancy

## Abstract

**Introduction:**

Birth preparedness and complication readiness (BP/CR) promotes timely access to skilled maternal and neonatal services, active preparation and decision-making for seeking health care to prevent any adverse outcomes. The aim was to assess level of male partner (MP) involvement in the birth plan, the attitude of the women towards maternal care and factors associated with BP/CR among obstetric referrals in rural Rwanda.

**Methods:**

This was a cross-sectional study among 350 pregnant women who were admitted as referrals at Ruhengeri hospital, between July 2015 and November 2015. Data was collected on socio-demographics, level of MP's participation in maternal health care and domestic activities, women's attitude towards involvement of men in maternal care and BP/CR. Any woman who arranged to have a birth companion, made a plan of where to deliver from, received health education on pregnancy and childbirth complications, saved money in case of pregnancy complication and had attended antenatal care (ANC) at least 4 times, was deemed as having made a birth plan.

**Results:**

The mean age was 27.7 years, while mean age of the spouse was 31.3 years. Majority of the women (n=193; 55.1%) and their spouse (n=208; 59.4%) had completed primary education. Men's role was found to be mainly in the area of financial support. The level of men ANC attendance was low (n=103; 29.4%), while 78 (22.3%) women were accompanied to the labor ward. However, there was a strong opposition to the physical presence of MP in the labor room (n=178; 50.9%). The main reason cited by women opposing MP presence is that it is against their culture for a man to witness the delivery of a baby. On multivariable analysis, maternal education level of secondary or higher adjusted odds ratio [AOR] 1.4 95% CI (1.8-2.6), formal occupation of spouse, AOR 2.4 95% CI (1.4-4.2) and personnel checked during ANC being community health worker AOR 2.2, 95% CI; (1.3-3.7) were associated with being well prepared.

**Conclusion:**

Male involvement in pregnancy and antenatal care is low. To increase men involvement in birth plan addressing cultural barriers and refraining care-givers and health facility policies towards family delivery is paramount.

## Introduction

Globally, about 287,000 mothers die each year because of problems related to pregnancy and childbirth; the majority of these (99%) occur in developing countries and, out of those, 51% occur in the sub-Saharan African region [1]. These deaths arise from pregnancy, childbirth or postpartum complications. A key strategy that can reduce the number of women dying from such complications is making a birth plan that constitutes birth-preparedness and complication readiness measures for pregnant women, their spouses and their families [1, 2]. Birth preparedness and complication readiness (BP/CR) is a comprehensive package aimed at promoting timely access to skilled maternal and neonatal services, promotes active preparation and decision making for delivery by pregnant women and their families [2]. This stems from the fact that every pregnant woman faces risk of sudden and unpredictable life threatening complications that could end in death or injury to herself or to her infant [3]. In sub-Saharan Africa, pregnancy and childbirth continue to be viewed solely as women issues [4–7], yet their spouse's have tremendous control over them either socially or economically. Evidence demonstrates that involving men in maternal health care has beneficial consequences like reduction in maternal morbidity and mortality due to a sufficient birth plan thus avoiding care-seeking delays from obstetric emergencies [8–11], increased institutional deliveries [5, 9, 10] and postnatal service utilization [9, 12]. These lead to a reduction in all the three phases of delay [10, 13] and therefore better birth outcomes. Despite the documented benefits, male involvement in maternal and child health (MCH) is still low in sub-Saharan Africa countries and in many settings, it is unthinkable that a male partner (MP) would be present in the labor room during delivery [8, 14]. The few supportive MP also have met unwelcoming, intimidating and unsupportive health systems, presenting a missed opportunity to embrace their commitment [15].

In Rwanda, several initiatives have been undertaken to increase access to skilled care at birth and emergency obstetric care for complications [16–18], some which encouraged MP with the aim of prioritizing women who come for antenatal care (ANC) with their partners [10, 19]. This resulted in high number of attending partners at 87% [20]. Nevertheless, the quality of ANC has remained largely sub optimal, missing opportunities to provide health education for the expectant couple, identify or address early signs of complications and turned away supportive husbands from further health consultation [13]. The aim was to assess level of MP involvement in the birth plan, the attitude of the women towards maternal care and factors associated with BP/CR among obstetric referrals in rural Rwanda.

## Methods

This was a cross sectional study among pregnant women who were admitted as referrals at Ruhengeri hospital located in Musanze district, Rwanda, between July 2015 and November 2015. According to the Population census 2012, Musanze district had a population of 368 267 inhabitants with a total fertility rate of 4.6 births per woman [21]. Literacy rate is 88.6% and 79.7% for men and females respectively [21]. Health insurance coverage is 85.1% and 65.3% of women are delivered by skilled birth attendants [21]. Uptake of postnatal care by skilled personnel was at 4.5% [21].

Ruhengeri hospital acts as a provincial referral hospital for high-risk obstetric cases and referrals from health centers and other district hospitals in the northern province. Medical services offered are covered by community-based health insurance (“*mutual d'sante*”) at contribution of an annual fee of RWF 3,000 (US$4.5), with a 10% surcharge for each episode of illness. In case of shortages of drug supplies, patients are requested to procure missing items from private pharmacies. During the study period, medical staff consisted of one specialist obstetrician, four medical officers, two intern doctors and 18 midwives running the unit.

The study included all pregnant women who presented as referrals at the maternity ward with willingness to consent and participate in this study. Participants were followed up to their discharge from hospital or death. Two trained research assistants identified participants while the principal investigator verified suitability for study inclusion. A pretested structured interview questionnaire was used for data collection, based on "*Monitoring BP/CR: tools and indicators for maternal and newborn health*" [3] and adapted according to local context and the objectives of the study. Data was collected using an interviewer-administered questionnaire on i) socio-demographic variables such as age, education level, marital status, employment status and personnel checked during ANC ii) Medical history on ANC, obstetric history, reasons for referral, mode of delivery and care received for obstetric complications were recorded; iii) Level of MP's participation in maternal health care and domestic activities and women's attitudes towards male partner involvement in BP/CR; iv) Data was also collected on BP/CR, based on the number of arrangements a woman had made, including arranged to have a birth companion or attendant during delivery, made a plan of where to deliver from, received health education on pregnancy and childbirth complications, saved money in case of pregnancy complication and attended antenatal care at least 4 times was deemed as having made a birth plan. Any woman who mentioned at least three of these four BP/CR steps was considered "well prepared". The remaining women were considered "less prepared".

The collected data were entered, coded, cleaned and analyzed using SPSS for Windows Version 18.0. First, simple frequency distributions were calculated. Comparisons of the proportion of women who are birth prepared by each category of the independent variables were done and statistical significance assessed using the Chi-square test. To identify factors associated with BP/CR, bivariate logistic regression were used. These results were expressed as the Odds Ratio (OR) and with 95% Confidence Interval (CI). Factors that were found to have a p-value of less than 0.2 in the bivariate analysis were then entered into multivariable logistic regression analysis to identify factors associated with BP/CR.

Ethical approval was obtained from the National Ethical Committee (N°582/RNEC/2013). Participants were recruited after getting informed consent, at a time when they had recovered from the acute obstetric complications that necessitated their admission.

## Results

During the study period, 350 women were recruited from 400 eligible participants; 26 were too unwell to consent, 15 were unable to complete the interview and 9 declined consent. Table 1 shows the socio-demographic and reproductive characteristics. Out of 350 women who were interviewed, 327 (93.4%) were married with their mean age and spouse being 27.7 and 31.3 years respectively. Majority of the women (n=193; 55.1%) and their spouse (n=208; 59.4%) had completed primary education. The level of birth preparedness among obstetric emergencies and their male partner's (MP) role (Table 2). Out of 350 women, 307 (87.7%) had made savings in case of pregnancy complications, 103 (29.4%) were accompanied by their husband during ANC, while 78 (22.3%) were accompanied to the labor ward by their husbands. Majority 203 (58.3%) of respondents reported a community health worker (CHW) had given health advice during current pregnancy.

**Table 1:** Socio-demographic and reproductive characteristics of respondents

Characteristics	Mean ± standard deviation
+Age (Years)	27.7 ± 6.0
+Spouse’s Age (Years)	31.3 ± 6.5
+Antenatal attendance (times)	2.9± 0.9
+Parity	2.6 ± 1.9
**Marital Status**	
In marital union	327 (93.4)
Not in marital union∞	23 (6.6)
**Education**	
None	114 (32.6)
Primary	193 (55.1)
Secondary and Above	43 (12.3)
**Occupation**	
Housewife	195 (55.7)
Business/Private employee	98 (28.0)
Government/salaried employee	57 (16.3)
**Spouse’s education**	
None	101 (28.9)
Primary	208 (59.4)
Secondary and Above	41 (11.7)
**Spouse’s occupation**	
Farming Business/Private employee	148 (42.3) 171 (48.9)
Government/salaried employee	31 (8.8)

+ Mean ± Standard Deviation

∞ Single, divorced and widowed

**Table 2:** Birth preparedness among obstetric emergencies and their husband’s role

†Characteristic	N (%)
**Making a birth plan**	
Arranged to have a birth companion or attendant during delivery	46 (13.1)
Made a plan of where to deliver from	55 (15.7)
Had health education on pregnancy and childbirth Complications	37 (10.6)
Save money in case of pregnancy complication	307 (87.7)
Attended antenatal care at least 4 times	79 (22.6)
**Husband’s role during antenatal care**	
Accompany wife	103 (29.4)
Take care of domestic chores when she is pregnant	135 (38.6)
Looked after the children at home	54 (15.4)
**Husband’s role during labor**	
Provided transport or gave money for transport	242 (69.1)
Accompanied her to labor unit	78 (22.3)
Stayed home with children	114 (32.6)
Bought baby’s clothes	223 (63.7)
Got some person to take care of the home during the mother’s absence	152 (43.4)
**Personnel checked during antenatal care**	
Health professional	147 (42.0)
Community health worker	203 (58.0)

† Respondents gave multiple responses

When women were asked about altitudes towards their MP role in maternal care, 310 women (88.6%) agreed their MP should accompany spouses for ANC, during delivery (n=278; 79.4%) and postnatal care (n=202; 57.7%) respectively. However, there was a strong opposition to the physical presence of MP in the labor room (n=178; 50.9%) (Table 3). The main reason cited by women opposing MP presence is that it is against their culture for a man to witness the delivery of a baby.

**Table 3:** Attitude of wives toward husband’s involvement in maternal care

Statements	N (%)
**Husband should escort wife for ANC**	
Strongly Agree	183 (52.3)
Agree	127 (36.3)
Disagree	24 (6.8)
Strongly disagree	16 (4.6)
**Husband should escort wife to hospital for delivery**	
Strongly Agree	161 (46.0)
Agree	117 (33.4)
Disagree	55 (15.7)
Strongly disagree	17 (4.9)
**Husband should escort wife to labor room**	
Strongly Agree	83 (23.7)
Agree	77 (22.0)
Doesn’t Know	12 (3.4)
Disagree	102 (29.1)
Strongly disagree	76 (21.7)
**Husband should escort wife for postnatal care**	
Strongly Agree	176 (50.3)
Agree	26 (7.4)
Doesn’t Know	26 (7.4)
Disagree	66 (18.9)
Strongly disagree	56 (16)

Table 4 shows the factors associated with birth preparedness and complication readiness. On binary analysis, Primigravidae compared to multigravidae OR 2.5 95% CI (1.4-4.3), ANC attendance of less than 4 times versus ≥ 4 times OR 1.9 95% CI (1.7-2.4), spouse's education level secondary or higher versus primary level or none, OR 1.3 95% CI (1.6-3.0), spouse's formal occupation versus informal occupation, OR 1.4 95% CI (1.8-2.5) and personnel checked during ANC by health professional versus CHWs OR 1.4 95% CI (1.2-1.9), were associated with being well prepared. On multivariable analysis, maternal education level of secondary or higher adjusted odds ratio [AOR] 1.4 95% CI (1.8-2.6), formal occupation of spouse, AOR 2.4 95% CI (1.4-4.2) and personnel checked during ANC being CHWs AOR 2.2, 95% CI; (1.3-3.7) were associated with being well prepared.

**Table 4:** Factors associated with birth preparedness and complication readiness

Characteristics	Birth preparedness		COR (95%CI)	a AOR (95%CI)
	Well (%)	Less (%)		
**Age (years)**				
< 20	8 (10.3)	27 (9.9)	0.9 (0.4-2.0)	0.7 (0.3-1.8)
21-29	48 (61.5)	140 (51.5)	1.0	
> 30	22 (28.2)	105 (38.6)	0.6 (0.3-1.1)	0.9 (0.5-1.6)
**Marital status**				
In marital union	70 (89.7)	257 (95.5)	1.0	
Not in marital union∞	8 (10.3)	15 (5.5)	2.0 (0.8-4.8)	1.9 (0.5-7.6)
**Occupation**				
Informal employee	66 (84.6)	229 (84.2)	1.0	
Formal employee	12 (15.4)	43 (15.8)	1.0 (0.4-1.9)	0.7 (0.3-2.1)
**Education**				
None or primary	68 (87.2)	239 (87.9)	1.0	
Secondary and above	10 (12.8)	33 (12.1)	1.2 (1.5-3.0)	1.4 (1.8-2.6)
**Spouse’s age (years)**				
< 24	9 (11.5)	33 (12.1)	1.0 (0.4-2.1)	0.3 (0.7-1.9)
≥ 25	69 (88.5)	239 (87.9)	1.0	
**Spouse’s occupation**				
Informal employee	58 (74.4)	261 (96.0)	1.0	
Formal employee	20 (25.6)	11 (4.0)	1.4 (1.8-2.5)	2.4 (1.4-4.2)
**Spouse’s education**				
None or Primary	49 (62.8)	260 (95.6)	1.0	
Secondary and above	29 (37.2)	12 (4.4)	1.3 (1.6-3.0)	0.7 (0.3-1.9)
**Parity**				
1	38 (48.7)	85 (31.3)	2.5 (1.4-4.3)	1.2 (0.8-1.9)
2-4	27 (34.6)	149 (54.8)	1.0	
≥ 5	13 (16.7)	38 (13.9)	1.9 (0.9-4.0)	0.7 (0.3-1.3)
**Travel time to health facility for ANC services**				
<1 hours	73 (93.6)	247 (90.8)	1.0	
≥ 1 hours	5 (6.4)	25 (9.2)	0.8 (0.3-1.8)	0.8 (0.3-2.1)
**Antenatal attendance**				
< 4 times	3 (3.8)	218 (80.1)	1	1
≥ 4 times	75 (96.2)	54 (19.9)	1.9 (1.7-2.4)	1.3 (0.8-2.1)
**Personnel checked during ANC**				
Health professional	22 (28.2)	125 (46.0)	1.0	1.0
Community health worker	56 (71.8)	147 (54.0)	1.4 (1.2-1.9)	2.2 (1.3-3.7)

a Adjusted for education of respondent, spouse’s education and occupation, parity, ANC attendance and personnel checked during ANC

∞ Single, divorced and widowed

## Discussion

It is assumed that men play little role in emergency obstetric care for their spouses. This study showed rather that women are well supported during pregnancy, as evidenced by financial support and assistance with household chores. The latter is an impressive departure from African traditional pattern of practice, where men do not do any household chores [4, 13].

Our study shows that male attendance during antenatal clinic (ANC) was low, despite Rwanda's well-intended policies. This could have been partly due to the initial intention on male partner (MP) involvement policy to prevent mother-to-child transmission turned into a strict requirement for male attendance, a factor that might have hindered women from attending alone [13]. Additionally, the requirement for having the MP accompany the woman on her first visit, but then restricting men's access after that point, denied men the opportunity to invest a continued interest in the pregnant woman's situation. This rate calls for improvement in MP involvement include: creating more awareness for male-targeted ANC education and support, design maternity wards which are able to accommodate men in terms of, couple-friendly infrastructure, or have separate male friendly waiting areas for MP who have accompanied their wives to access maternal health care [14, 22, 23].

Our findings are in agreement with others [8, 24] that women who were well prepared are more likely to be accompanied by the spouses to health facilities during ANC and to the labor ward during labor. They were also more likely to report more support from spouses in looking after children or take care of domestic chores during pregnancy. This finding suggests educating MP about BP/CR during ANC visits might increase their involvement in birth plan. A two recent systematic review that looked at interventions for male involvement and maternal health outcomes found that the MP involvement improved birth preparedness and was associated with better labor, delivery and postpartum follow up outcomes [10, 23].

Our study shows that being well prepared was associated with maternal education level and occupation of spouse. Educated women have better pregnancy outcome compared with uneducated women, possibly due to better understanding of health messages acquired from mass media, and prior exposure to educational information, factors that influence behavior change or financial stability to make the necessary decisions in case of obstetric emergencies [8, 10, 22]. When women were asked what they would do when the husband was not around in case of emergency obstetric care, majority replied they called upon community health worker (CHWs) who advised or escorted them to skilled birth attendant, contrary to what other authors have reported that approval was needed first directly or indirectly from the MP or a family member respectively [8, 9, 11]. This shows high the level of trust expectant mothers from rural Rwanda have for CHWs, who previously were considered well informed on matters relating to BP/CR and in cases of an emergency would be called on their mobile phone to offer assistance [16].

This study has some limitations. Our results can not be generalizable due to the fact that this was a cross sectional hospital-based among referred pregnant women, temporal relationship could not be established. The presence of obstetric complications may have influenced the acquisition and therefore availability of a BP/CR. Indeed, some women may recall or provide information about the BP/CR selectively, depending on the delivery experience or pregnancy outcome. The questions about the BP/CR and their husband's role in the latter were asked after delivery or after complications had occurred, which may have created some bias. Ideally, they should be asked before delivery. However, this was the objective of the study, that was assessing husband's role in birth plan and complication readiness. There is no way one can verify that the responses were not the socially desirable responses, more so considering that the interviews were conducted at the a health facility. The interviews were conducted in absence of spouses to avoid biased responses. Despite the limitations, we believe the study provides relevant information on BP/CR for women in rural areas, and identify missed opportunities for interventions to improve emergency obstetric care. Future longitudinal studies would be a good option to collect dynamic information on male roles in BP/CR.

## Conclusion

Male involvement in pregnancy and antenatal care is low. The importance of prenatal advice by CHWs is emphasized. Education at all levels is desirable, due to all its positive associations, with specific education on family participation. To increase men involvement in birth plan addressing cultural barriers and refraining care-givers and health facility policies towards family delivery is paramount.

### What is known about this topic

- In most African countries, planning for pregnancy and childbirth are largely regarded as exclusively women's concerns, and while men may accompany their partners to antenatal or postnatal care, they are usually not expected to attend the labor or birth of their baby;
- Making informed decisions about maternal health requires the male partner to understand the importance of ANC, planning for skilled birth attendance and birth plan to avoid the risks associated with pregnancy and delivery;
- Several interventions have been suggested for supporting male partner involvement in pregnancy and safe delivery, but no assessment has been made in terms of their effectiveness.

### What this study adds

- Male involvement in pregnancy and labor care can improve maternal and new born outcomes;
- Having an educated mother who has an economically stable spouse are necessary ingredients for well preparedness for pregnancy and delivery;
- To increase men involvement in birth plan addressing cultural barriers and refraining care-givers and health facility policies towards family delivery is paramount.
